# Effects of gestational diabetes mellitus and diabetes mellitus on lipid profile, antioxidants, hormones and electrolytes status in a population of Nigerian women

**DOI:** 10.1186/s40842-024-00206-4

**Published:** 2025-01-21

**Authors:** Moses Orimoloye Akinjiyan, Adeniran Samuel Atiba, Modupe Fisayo Asaolu

**Affiliations:** 1 Medical Biochemistry, School of Basic Medical Sciences, Federal University of Technology, Akure, Ondo State Nigeria; 2 Department of Chemical Pathology, Ekiti State University Teaching Hospital, Ado-Ekiti, Ekiti State Nigeria; 3 Department of Biochemistry, Ekiti State University, Ado-Ekiti, Ekiti State Nigeria

**Keywords:** Gestational diabetes mellitus, Diabetes mellitus, Hormone, Electrolytes, Antioxidants, Lipid profile

## Abstract

**Aim:**

Gestational diabetes mellitus (GDM) cases are rising globally. This research examined the effects of GDM and diabetes mellitus whose hallmark is hyperglycemia on hormones, lipid profiles, electrolytes and antioxidants in freshly diagnosed women attending teaching hospitals in Ekiti State, Nigeria.

**Methods:**

The cross-sectional design followed convenience sampling in four groups (*n* = 50): gestational diabetes women (GDM), normal pregnant women (NP), diabetes nonpregnant women (DM) and nondiabetes nonpregnant women (ND-NP). Blood samples were collected from fasting subjects through antecubital fossa. BMI, FPG, TC, TG, LDL-C, HDL-C, sodium, potassium and bicarbonate ions, MDA and antioxidants were investigated using standard procedures and spectrophotometer. Results were analysed using one-way ANOVA and *p* < 0.05 was used as significant difference.

**Results:**

There was significant (*p* < 0.05) increase in FPG of GDM and DM (> 5.56 mmol/l) subjects compared to NP and ND-NP subjects. TC, TG, HDL-C and LDL-C were elevated in GDM and DM groups compared to NP and ND-NP groups. Sodium ion concentration was significantly (*p*˂0.05) elevated in ND-NP group relative to GDM and DM groups. There was significant (*p* < 0.05) increase in MDA levels in DM and NP groups compared to ND-NP. Superoxide dismutase activity was significantly (*p* < 0.05) greater in ND-NP than in DM and NP. Progesterone level was greater in GDM group than in other groups. The FSH concentration was lower in the GDM and NP groups than in the DM and ND-NP groups, and prolactin concentration was greater in the GDM group than in the NP.

**Conclusion:**

This study suggested that GDM and DM could result in hormonal and electrolyte imbalances, elevated lipid profiles, and reduced antioxidant levels.

**Supplementary Information:**

The online version contains supplementary material available at 10.1186/s40842-024-00206-4.

## Introduction

Major public health challenges include gestational diabetes mellitus (GDM) and diabetes mellitus (DM) among others. Women who develop type 1 or type 2 DM during pregnancy confirmed by oral glucose tolerance test are referred to have GDM [1]. GDM characterized by elevated blood glucose levels complicates approximately seven per cent of pregnancies overall, accounting for more than 200,000 pregnancies annually, resulting in maternal death, macrosomia, and virginal tears, among others. Between 3 and 10% of expectant mothers develop GDM, making it one of the most common problems associated with gestation [2]. Depending on the population under study and the diagnostic procedures employed, the incidence could also vary from approximately 2 to 14% of all gestations [3]. Due to ethnicity and ethnic heterogeneity within the various populations studied, global GDM prevalence rates exhibit significant variances. This complexity is further compounded by the various screening and diagnostic criteria employed. In Africa, the prevalence of GDM in the antenatal population is low and varies; the reported prevalence is 3.7% of expectant mothers while about 13.9% prevalence was found in Nigeria [3, 4].

DM patients whose symptoms are persistent high blood glucose can be type 1, type 2 or gestational [5]. The estimated worldwide incidence of diabetes in 2010 was approximately 300 million, and this value is expected to increase to an estimated 440 million by 2030 [6]. Owing to this prevalence of DM worldwide, the World Health Organization (WHO) predicted an ever-increasing number of DM cases worldwide. By 2025, the incidence of DM in Africa is expected to increase to 15 million. With roughly millions of cases, Nigeria has the highest number of diabetes cases in Africa [7].

Oxidative stress, a type of cell damage caused by free radicals, can be prevented and managed by antioxidants such as vitamins, catalase (CAT), superoxide dismutase (SOD) and reduced glutathione (GSH) [8]. The need for antioxidants becomes even more important with increased exposure to free radicals during pregnancy, which may lead to conditions such as DM and GDM. DM and GDM have been associated with reactive oxygen species (ROS) leading to oxidative stress [8]. Failure of the body’s intracellular antioxidant system to compensate for these ROS increases oxidative stress, resulting in the activation of stress-sensitive intracellular signalling pathways and ultimately cellular damage and disorders in biomolecule metabolism which leads to the pathogenesis of DM.

Biomolecules such as lipids can be produced by the body or acquired through the diet. These components include phospholipids, triacylglycerides (TGs), cholesterol, and sphingolipids, which are important membrane constituents [9]. The unique carriers known as lipoproteins (low-density and high-density lipoproteins) aid in the movement of these lipids throughout the body. The body uses lipids obtained from food that are enzymatically digested by lipases into fatty acids and glycerol, while any surplus lipids are stored as TG in fat, muscle, and liver cells (adipocytes, osteocytes, and hepatocytes, respectively) [10]. Cholesterols are a starting point of steroid hormones such as progesterone, an essential pregnancy hormone, and they aid in maintaining the integrity of cell membranes in both solid and fluid states [11].

Chemicals called hormones function in the human body as messenger molecules. Progesterone is a known pregnancy hormone, although high levels of progesterone have been linked with insulin resistance in pregnancy. Disturbances in female hormones such as follicle stimulation and prolactin may be associated with GDM [12].

Sodium ions (Na^+^) and potassium ions (K^+^) are electrolytes that help in the transportation of glucose in and out of cells via sodium-potassium pumps which are located on the membranes [13]. Bicarbonate ion (HCO3^−^) is a polyatomic anion that is produced in the stomach and is essential for the digestion of food materials such as glucose. A carbonic anhydrase-catalyzed reaction between carbon IV oxide and water can also occur during the metabolism [14]. This work investigated if GDM and DM have effects on hormones, lipid profiles, electrolytes and antioxidants among a population of Nigerian women.

## Materials and methods

The subjects (200) were gestational diabetes, diabetes, pregnant and nondiabetes nonpregnant women attending the antenatal and outpatient department (OPD) clinic of EKSUTH, Ado-Ekiti and FTH, Ido-Ekiti and a few basic health centres in the Ado-Ekiti from 2016 to 2017. It is a cross-sectional design and a convenience method of sampling was used for the grouping as follows:

- Group 1 included fifty (50) women with gestational diabetes;
- Group 2 included fifty (50) normal pregnant women;
- Group 3 included fifty (50) diabetes nonpregnant women and.
- Group 4 included fifty (50) nonpregnant and nondiabetes women.

Pregnant and non-pregnant women with fasting plasma glucose greater than 5.56 mmol/l were diagnosed with GDM (group 1) and DM (group 3) respectively after 1 h and 2 h, 75 g oral glucose tolerance test was done.

The following formula was used for the sample size:

$$N=\frac{t2\;P\;(1-P)}{m2}$$

Where;

𝑁: sample size,

𝑡: confidence interval of 95% (standard value of 1.96),

𝑃: prevalence rate (3.7%),

𝑚: margin of error (standard value of 0.05).

Hence 𝑁 = 51.9. An approximate sample size of 50 per group and 200 samples in total.

### Exclusion criteria

Subjects who had not fasted (for approximately 10 h overnight) were excluded. Women with known (based on interactions) renal diseases, hypertension or malaria concurrent with pregnancy, were also excluded from the GDM and DM subjects. This is done because these conditions could influence the biochemical parameters being examined.

### Data and sample collection

The participants’ anthropometric measurements, including height and weight, were taken using standiometers and weighing balances, respectively. The equation used for calculating body mass index (BMI) is mass (kg)/height^2^ (m^2^) [15]. A sphygmomanometer was used to measure blood pressure. Blood samples (approximately 8 ml) were collected through venepuncture from the antecubital fossa after cleaning the area with a methylated spirit, and approximately 3 ml and 5 ml were dispensed into fluoride oxalate and plain specimen bottles, respectively. The pregnant women were loaded with 75 g of glucose dissolved in a volume of water and found to be comfortable for the patient to finish within the next five minutes. Samples (approximately 3 ml) were further transferred to fluoride oxalate bottles after 1 and 2 hours of glucose solution treatment. Pregnant and non-pregnant women with fasting plasma glucose greater than 5.56 mmol/l were diagnosed with GDM and DM respectively.

### Sample analysis

All samples at each stage were centrifuged at 1200 rpm for 11 min; plasma and serum were collected into plain bottles, appropriately labelled and stored at -20 °C until the analyses were performed using standard methods with chemicals of high analytical grade. The plasma glucose was measured immediately using the glucose oxidase method [16]. The glucose levels of all the subjects were evaluated, and further analyses were performed on some of them.

#### Antioxidant and lipid peroxidation analysis

Lipid peroxidation (malonyldialdehyde) and antioxidants (SOD, GSH and CAT) levels were determined using standard spectrophotometric procedures as described previously by Ayo et al. [17].

##### Lipid peroxidation

Samples (0.5 ml) were added to 0.5 ml of phosphate buffer (0.1 M, pH 8.0) and 0.5 ml of 24% TCA. The resulting mixture was incubated at room temperature for 10 min, followed by centrifugation at 2000 rpm for 20 min. To 1 ml of supernatant was added 0.25 ml of 0.33% TBA in 20% acetic acid and the resulting mixture was boiled at 95 ^o^C for 1 h. The resulting pink colored product was cool and absorbance was read at 532 nm.

##### Superoxide dismutase

To 200 µL of the lysate, 2.5 ml of 75 mM of Tris–HCl buffer (pH 8.2), 30 mM EDTA and 300 µL of 2 mM of pyrogallol were added. An increase in absorbance was recorded at 420 nm for 3 min by spectrophotometer. One unit of enzyme activity is 50% inhibition of the rate of autooxidation of pyrogallol as determined by a change in absorbance/min at 420 nm. The activity of SOD is expressed as units/mg protein.

##### Catalase

Fifty microlitres (50 µl) of the sample is added to a cuvette containing 450 µl of phosphate buffer (0.1 M, pH 7.4) and 500 µl of 20 mM H_2_O_2_. Catalase activity is measured at 240 nm for 1 min using a spectrophotometer. The molar extinction coefficient of H_2_O_2_, 43.6 M cm^−1^ was used to determine the catalase activity. One unit of activity is equal to 1 mmol of H_2_O_2_ degraded per minute and is expressed as units per milligram of protein.

##### Reduced glutathione

1 ml of the supernatant from the centrifuged tube was taken 0.5 ml of Ellman’s reagent (10mM) and 2 ml of phosphate buffer (0.2 M, pH 8.0) were added. The yellow colour developed was read at 412 nm with a blank containing 3.5 ml of phosphate buffer. A series of standards were also treated similarly.

$$\mathrm{GSH}\;\mathrm{concentration}\;=\;\mathrm{Absorbance}\;\mathrm{of}\;\mathrm{test}\;\times\;310.4\;(\mathrm{factor})$$

#### Lipid profile

Estimates of the concentrations of triglycerides, high- and low-density lipoprotein cholesterol, and serum total cholesterol were made following the spectrophotometric methods of Aberare et al. [18].

##### Triglycerides

Ten microliters (10 µl) of serum, control and standard samples were pipette into different test tubes containing 1 ml of triglyceride reagent and mixed. The mixture was incubated for 10 min and the absorbance was read at 520 nm wavelength.

$$\begin{array}{lc}\mathrm{Concentration}\;\mathrm{of}\;\mathrm{Serum}\;\;\;\;=&\underline{\mathrm{Absorbance}\;\mathrm{of}\;\mathrm{test}\;\;\mathrm X\;\;\mathrm{Concentration}\;\mathrm{of}\;\mathrm{standard}}\\\mathrm{Triacylglyceride}\;(\mathrm{mmol}/\mathrm l)&\mathrm{Absorbance}\;\mathrm{of}\;\mathrm{standard}\end{array}$$

##### Total cholesterol

Serum (10 µl), control and standard samples were pipette into different test tubes containing 1 ml of cholesterol reagent and mixed. The mixture was incubated for 10 min and read at 520 nm.

$$\begin{array}{lc}\mathrm{Concentration}\;\mathrm{of}\;\mathrm{Serum}\;\;\;\;=&\underline{\mathrm{Absorbance}\;\mathrm{of}\;\mathrm{test}\;\;\mathrm X\;\;\mathrm{Concentration}\;\mathrm{of}\;\mathrm{standar}}\mathrm d\\\mathrm{Cholesterol}\;(\mathrm{mmol}/\mathrm l)&\mathrm{Absorbance}\;\mathrm{of}\;\mathrm{standard}\end{array}$$

##### High-density lipoprotein cholesterol

Five hundred microlitres (500 µl) of serum, control and standard samples were pipette into different plastic tubes containing 1 ml of phosphotungstic acid and centrifuged for ten minutes at 4000 rpm. One hundred microliters (100 µl) of serum, control and standard supernatant were pipette into different test tubes containing 1 ml cholesterol reagent and mixed. The mixture was incubated for 10 min and read at 520 nm.

$$\begin{array}{lc}\mathrm{Concentration}\;\mathrm{of}\;\mathrm{Serum}\;\;\;\;=\;&\underline{\mathrm{Absorbance}\;\mathrm{of}\;\mathrm{test}\;\;\mathrm X\;\;\mathrm{Concentration}\;\mathrm{of}\;\mathrm{standard}}\\\mathrm{High}-\mathrm{density}\;\mathrm{lipoprotein}\;\mathrm{cholesterol}\;(\mathrm{mmol}/\mathrm l)&\mathrm{Absorbance}\;\mathrm{of}\;\mathrm{standard}\end{array}$$

##### Low-density lipoprotein cholesterol

Serum Low-density lipoprotein-cholesterol was analysed mathematically using Friedewald Equation.

$$LDL-C\,(mmol/1)=TC-(HDL-C)+\frac{TG}{2.2}$$

Where TC = Total Cholesterol, HDL-C = high density lipoprotein-cholesterol and TG = Triacylglyceride.

#### Determination of electrolyte concentrations

Serum concentrations of sodium and potassium were determined using the flame photometry method as described by Garcia et al. [19]. Serum and standard [containing 58.5 g/L of NaCl and 7.46 g/L of KCl of equal volume] of 0.1 ml was pipetted into respective electrolyte bottles (of test and standard) and diluted with distilled water (10 ml). The flame photometer was turned on and blanked with distilled water. The required electrolyte filter was selected, after which the nebulizer was introduced into the diluted standard and test. The concentration of sodium and potassium in the serum samples and standard in mmol/L was visualised through the read-out device of the flame photometer.

The serum bicarbonate concentrations were estimated as described by Al-Kindi [20]. After standardizing 0.01 M NaOH and 0.01 M HCl (1 ml 0.01 M NaOH neutralising 0.01 M HCl), 100 µl of serum sample was dispensed into a titrant reservoir followed by the addition of 1 ml of 0.01 N HCl and 1 drop of phenol red indicator. One millilitre pipette was filled with 0.01 N NaOH and titrated with the mixture in the reservoir to the first permanent pinkish-red endpoint as titre value.

$$\begin{array}{c}\mathrm{Number}\;\mathrm{of}\;\mathrm{moles}\ 0.01\mathrm M{\;\mathrm{HCO}}_{3^-}=\;\;\;\mathrm{Number}\;\mathrm{of}\;\mathrm{moles}\;{1\mathrm{cm}}^3\;0.01\;\mathrm M\;\mathrm{HC}\\\mathrm C\;{\mathrm{HCO}}_{3^-}\times\mathrm V\;{\mathrm{HCO}}_{3^-}=\mathrm C\;\mathrm{HCl}\;\times\;{\mathrm V}_{\mathrm{HCI}}\end{array}$$

#### Determination of hormone concentrations

The quantitative determination of FSH, prolactin and progesterone levels was according to a solid-phase enzyme-linked immunosorbent assay as outlined by Porstmann and Kiessig [21]. Standards, specimens and controls (50 µl) were dispensed into appropriate wells. Enzyme conjugate reagent (100 µl) was added into each well and thoroughly mixed for 30 s followed by incubation at room temperature (about 25 ^o^C) for 60 min. The microwells were rinsed and flicked with washing buffer 5 times. The well was firmly tapped against paper to remove all residual water droplets. TMB solution (100 µl) was dispensed into each well and it was gently mixed for 5 s followed by incubation in the dark at room temperature for 20 min. The reaction was stopped by adding 100 µl of stop solution to each well and gently mixed for 30 s. The absorbance for each was read at 450 nm wavelength with a microtiter well reader within 15 min. The absorbance value for each specimen was used to determine the corresponding concentration of each hormone from the standard curve.

### Statistical analyses

One-way analysis of variance and post-hoc Duncan test were used to analyse the study’s data, which were then expressed as mean ± standard deviation, with *p* < 0.05 regarded as a significant difference. All analyses were performed with version 20 of the Statistical Package for Social Sciences (SPSS) [17].

## Results

### Anthropometric parameters of gestational diabetes, normal pregnant, diabetes nonpregnant and nondiabetes nonpregnant women

The mean body mass indices (BMIs) of gestational diabetes (GDM) (32.38 ± 4.25 kgm^−2^) and diabetes (DM) women (31.95 ± 12.48 kgm^−2^) increased compared to those of normal pregnant (27.85 ± 8.58 kgm^−2^) and nondiabetes nonpregnant (25.24 ± 3.30 kgm^−2^) women, as shown in Table 1. The diabetes group (84 ± 38.18 kg) had the greatest weight, while the gestational diabetes group had the least height (1.55 ± 0.04 m) compared to the other groups. The average and standard deviation of the gestational age differed little between the GDM (17.76 ± 5.46 weeks) and DM (17.62 ± 3.33) groups. The systolic and diastolic blood pressures of diabetes women (164 ± 45.05 and 88.00 ± 10.95 mmHg) were significantly greater (*p* < 0.05) than those of normal pregnant (109.29 ± 11.41 and 67.86 ± 5.88 mmHg) and nondiabetes nonpregnant women (108 ± 15.98 and 73.25 ± 6.29 mmHg).

**Table 1:** Anthropometric parameters of gestational diabetes, normal pregnant, diabetes nonpregnant and nondiabetes nonpregnant women

Anthropometric parameters	Gestational diabetes	Normal pregnant	Diabetes nonpregnant	Nondiabetes, nonpregnant
Age(yrs)	33.78 ± 2.59^b^	27.00 ± 3.87^a^	41.86 ± 10.17^c^	38.25 ± 5.85^b^
Body weight(kg)	77.4 ± 8.50^ab^	66.92 ± 14.05^ab^	84.00 ± 38.18^b^	56.80 ± 12.40^a^
Height(m)	1.55 ± 0.04^a^	1.59 ± 0.07^a^	1.61 ± 0.06^a^	1.62 ± 0.07^a^
Body Mass Index (BMI) (kgm^−2^)	32.38 ± 4.25^a^	27.85 ± 8.58^a^	31.95 ± 12.48^a^	25.24 ± 3.30^a^
Gestational Age (weeks)	17.76 ± 5.46	17.62 ± 3.33		
Systolic Blood pressure (mmHg)	112.31 ± 12.35^a^	109.29 ± 11.41^a^	164 ± 45.05^b^	108 ± 15.98^a^
Diastolic blood pressure (mmHg)	72.34 ± 9.27^a^	67.86 ± 5.88^a^	88.00 ± 10.95^b^	73.25 ± 6.29^a^

The data are shown as means ± standard deviations (SDs) of the participants’ anthropometric parameters, with varying superscripts (a, b and c) denoting significant differences at *p* ˂ 0.05

### Glucose and lipid profiles of gestational diabetes, normal pregnant, diabetes nonpregnant and nondiabetes nonpregnant women

As shown in Table 2, the fasting plasma glucose levels of gestational diabetes (6.05 ± 1.32 mmol/l) and diabetes women (9.80 ± 4.56 mmol/l) after 75 g oral glucose tolerance test was done were significantly greater (*p* < 0.05) than those of normal pregnant (3.63 ± 0.58 mmol/l) and nondiabetes nonpregnant women (4.27 ± 0.63 mmol/l). The 1-hour postprandial HPP (1 HPP) of gestational diabetes women (6.94 ± 2.38 mmol/l) was greater than that of normal pregnant women (5.02 ± 1.23 mmol/l); likewise, the 2 HPP values were 6.39 ± 2.42 mmol/l and 4.82 ± 1.07 mmol/l, respectively.

The serum triglyceride (TG) level was significantly greater (*P* < 0.05) in gestational diabetes women (1.97 ± 0.85 mmol/l) than in normal pregnant (1.21 ± 0.29 mmol/l) and nondiabetes nonpregnant women (1.18 ± 0.33 mmol/l). Low-density lipoprotein cholesterol (LDL-C) was significantly greater (*p* < 0.05) in the diabetes group (3.93 ± 1.11 mmol/l) than in the normal pregnant (2.51 ± 0.80 mmol/l) and nondiabetes nonpregnant (2.30 ± 0.62 mmol/l) groups. Total cholesterol (TC) was significantly elevated (*p* < 0.05) in diabetes women (5.69 ± 0.83 mmol/l) compared to that in normal pregnant women (4.71 ± 0.89 mmol/l).

**Table 2:** Glucose and lipid profiles of gestational diabetes, normal pregnant, diabetes nonpregnant and nondiabetes nonpregnant women

Biochemical parameter (mmol/l)	Gestational diabetes	Normal pregnant	Diabetes nonpregnant	Nondiabetes, nonpregnant
FPG	6.05 ± 1.32^b^	3.63 ± 0.58^a^	9.80 ± 4.56^c^	4.27 ± 0.63^a^
1HPP	6.94 ± 2.38	5.02 ± 1.23		
2HPP	6.39 ± 2.42	4.82 ± 1.07		
TG	1.97 ± 0.85^b^	1.21 ± 0.29^a^	1.54 ± 0.70^ab^	1.18 ± 0.33^a^
TC	4.98 ± 1.31^ab^	4.71 ± 0.89^a^	5.69 ± 0.83^b^	4.88 ± 1.14^ab^
HDL-C	2.09 ± 0.50^b^	1.67 ± 0.28^a^	1.62 ± 0.75^a^	1.35 ± 0.45^a^
LDL-C	2.64 ± 0.89^a^	2.51 ± 0.80^a^	3.93 ± 1.11^b^	2.30 ± 0.62^a^

Data are expressed as the mean ± SD of participants’ data with varying superscripts (a, b and c) denoting significant differences at *p* ˂ 0.05

*Keywords*: *FPG *Fasting plasma glucose, *1 HPP and 2 HPP*  1 h and 2 HPP postprandial, *TG *Triglycerides, *TC *Total cholesterol, *HDL-C and LDLC *High-density and low-density lipoprotein cholesterol

### Antioxidant activity and serum concentrations of malonyldialdehyde in gestational diabetes, normal pregnant, diabetes non-pregnant and non-diabetes non-pregnant women

The serum GSH concentration was significantly (*p* < 0.05) lower in normal pregnant (0.32 ± 0.04 mmol/l), diabetes (0.45 ± 0.12 mmol/l), and gestational diabetes (0.45 ± 0.08 mmol/l) women than in nondiabetes nonpregnant (0.50 ± 0.17 mmol/l) women (Table 3). CAT activity increased in nondiabetes nonpregnant women (2.06 ± 1.86 mU/l) but decreased nonsignificantly (*p* > 0.05) in gestational diabetes (1.29 ± 0.56 mU/l), normal pregnant (1.28 ± 0.70 mU/l), and diabetes (1.20 ± 1.03 mU/l) women. Compared to nondiabetes nonpregnant women (1.42 ± 0.36 U/ml), diabetes nonpregnant women (0.90 ± 0.14 U/ml) had significantly lower mean SOD activity (*p* < 0.05). The activity of vitamin E, a nonenzymatic antioxidant, decreased in gestational diabetes (16.33 ± 1.45 mg/dl) and diabetes nonpregnant (16.62 ± 15.27 mg/dl) women but increased in nondiabetes, nonpregnant (18.48 ± 12.9 mg/dl) women.

**Table 3:** Antioxidant activities and serum concentrations of Malonyldialdehyde in gestational diabetes, normal pregnant, diabetes nonpregnant and nondiabetes nonpregnant women

Antioxidants	Gestational diabetes	Normal pregnant	Diabetes nonpregnant	Nondiabetes, nonpregnant
MDA(µmol/l)	4.62 ± 0.84^ab^	5.34 ± 1.19^bc^	5.74 ± 1.17^c^	4.11 ± 1.02^a^
GSH(mmol/l)	0.45 ± 0.08^b^	0.32 ± 0.04^a^	0.43 ± 0.12^b^	0.50 ± 0.17^b^
SOD (U/ml)	1.34 ± 0.42^bc^	1.07 ± 0.41^ab^	0.90 ± 0.14^a^	1.42 ± 0.36^c^
CAT (mU/l)	1.29 ± 0.56^a^	1.28 ± 0.70^a^	1.20 ± 1.03^a^	2.06 ± 1.86^a^
Vit C (g/l)	0.38 ± 0.17^a^	0.45 ± 0.22^a^	0.44 ± 0.17^a^	0.21 ± 0.05^a^
Vit E (mg/dl)	16.33 ± 1.45^a^	17.24 ± 14.08^a^	16.62 ± 15.27^a^	18.48 ± 12.9^a^

The data are expressed as the means ± SDs of the participants’ antioxidant activities and malonyldialdehyde concentrations, with varying superscripts (a, b and c) denoting significant differences at *p* ˂ 0.05

*Keywords*: *GSH *reduced glutathione, *CAT *catalase, *MDA *malonyl dialdehyde, *SOD *superoxide dismutase, *Vit *vitamin

### Serum concentrations of electrolytes in gestational diabetes, normal pregnant, diabetes non-pregnant and non-diabetes non-pregnant women

The potassium ion (K^+^) concentration in the serum was significantly lower (*p* < 0.05) in women with GDM (3.54 ± 0.16 mmol/l) than in nondiabetes nonpregnant individuals (4.20 ± 0.42 mmol/l) (Table 4). Compared to those in normal pregnant subjects (132.33 ± 2.23 mmol/l) and nondiabetes nonpregnant subjects (134.67 ± 11.91 mmol/l), the sodium ion (Na^+^) level was significantly lower (*p* > 0.05) in GDM (128.83 ± 3.51 mmol/l) and diabetes (129.55 ± 7.19 mmol/l) women. Compared to diabetes women (22.73 ± 1.27 mmol/l), nondiabetes nonpregnant women had a significantly greater serum bicarbonate ion concentration (*p* > 0.05). The level of copper was not significantly different across the groups.

**Table 4:** Serum concentrations of Electrolytes in gestational diabetes, normal pregnant, diabetes nonpregnant and nondiabetes nonpregnant women

Biochemical parameter (mmol/l)	Gestational diabetes	Normal pregnant	Diabetes nonpregnant	Nondiabetes, nonpregnant
Na^+^	128.83 ± 3.51^a^	132.33 ± 2.23^a^	129.55 ± 7.19^a^	134.67 ± 11.91^a^
K^+^	3.54 ± 0.16^a^	3.71 ± 0.25^ab^	3.87 ± 0.20^bc^	4.20 ± 0.42^c^
HCO_3_^−^	23.50 ± 1.98^ab^	23.60 ± 1.18^ab^	22.73 ± 1.27^a^	24.07 ± 1.28^b^
Cu	0.20 ± 0.07^a^	0.18 ± 0.03^a^	0.17 ± 0.07^a^	0.16 ± 0.10^a^

The data are expressed as the means ± SDs of the participants’ electrolyte levels, while the superscripts (a, b and c) denote significant differences. P ˂ 0.05 was considered statistically significant

*Keywords*: *Na+, K+ and HCO3− *sodium, potassium and bicarbonate ions, respectively

### Serum concentrations of hormones in gestational diabetes, normal pregnant, diabetes non-pregnant and non-diabetes non-pregnant patients

Table 5 shows that the progesterone concentration in GDM women was markedly greater than that in normal pregnant (4.95 ± 2.90 ng/ml), diabetes nonpregnant (4.35 ± 2.90 ng/ml), and nondiabetes nonpregnant (2.60 ± 2.97 ng/ml) women. The follicle-stimulating hormone (FSH) concentration was lower in GDM (1.43 ± 0.63 mIU/ml) and normal pregnant (1.75 ± 0.49 mIU/ml) subjects than in nondiabetes nonpregnant (11.25 ± 2.47 mIU/ml) and diabetes (12.80 ± 3.58 mIU/ml) subjects. In GDM patients, prolactin levels were marginally greater than those in healthy pregnant women (5.15 ± 5.44 ng/ml).

**Table 5:** Serum Concentrations of Hormones in gestational diabetes, normal pregnant, diabetes nonpregnant and nondiabetes nonpregnant women

Hormones	Gestational diabetes	Normal pregnant	Diabetes nonpregnant	Nondiabetes, nonpregnant
Progesterone (ng/ml)	15.90 ± 9.05^a^	4.95 ± 2.90^b^	4.35 ± 2.90^b^	2.60 ± 2.97^a^
Prolactin (ng/ml)	11.0 ± 2.83^a^	5.15 ± 5.44^b^	6.27 ± 5.71^b^	12.95 ± 7.0^a^
Follicle-stimulating hormone(mIU/ml)	1.43 ± 0.63^a^	1.75 ± 0.49^a^	12.80 ± 3.58^b^	11.25 ± 2.47^b^

Values are expressed as the mean ± SD of patients’ serum hormone concentrations, while superscripts (a, b or c) are significantly different at *p *˂ 0.05

## Discussion

Table 1 shows there is a significant (*p* < 0.05) increase in blood pressure in diabetes women relative to nondiabetes nonpregnant women. This could be a result of hyperglycemic conditions in the diabetic state; increased blood pressure (BP) and insulin resistance, which cause diabetes, have also been reported [22]. This also suggests that increased BP in pregnancy state could be a pointer to GDM and managing BP could help put GDM in check. The mean gestational age of GDM women (17.76 weeks) and normal pregnant women (17.62 weeks) did not significantly differ (*p* < 0.05). This finding demonstrated that the development of GDM is primarily not determined by gestational age but rather could be caused by physiological changes, genetics, diet (wish could be based on socioeconomic status), physical activity or environmental factors that cause disturbance in glucose metabolism resulting in the insulin resistance (IR) and elevated blood sugar observed in the pregnant individuals with GDM (Table 2). This finding is similar to that of Ploughs et al., who reported that GDM could be a function of heredity [23]. Table 1 also shows an increase in the body weight of diabetes women compared to that of nonpregnant nondiabetes women. The buildup of adipose tissues or fats, such as visceral fats, is typically what leads to excess weight (liver, muscle and other organs). Environmental factors such as overfeeding or genetics may be responsible for this fat buildup as also reported by Qualls-Creekmore et al. [24]. Overweight visceral fat, especially in the waist or lower abdomen region, has been linked to an elevated chance of IR, which increases the risk of obesity, hyperglycemia, hyperinsulinemia, and diabetes [25]. Hyperlipidemia in hyperglycemic patients has also been reported [26]. Increased physical activity and limiting fatty food intake could help reduce lipid buildup, and thus manage both DM and GDM cases.

The significant increase (*p* < 0.05) observed in the mean fasting plasma glucose of gestational diabetes and diabetes women compared to that of normal pregnant and nondiabetes nonpregnant women, as shown in Table 2, may be linked to the fact that IR does not allow glucose to enter cells. This prevents glucose from being used as energy or its excess from being stored as glycogen, thus making it more than normal in the blood. Jing et al. [27] also reported that IR is associated with increased blood sugar.

The serum concentration of malonyldialdehyde (MDA), an aftermath of lipid peroxidation, was significantly greater (*p* < 0.05) in normal pregnant and diabetes patients than in nondiabetes nonpregnant women (Table 3). Hyperglycaemia-induced oxidative stress, which is a result of elevated mitochondrial reactive oxygen species (ROS), excessive nonenzymatic glycosylated protein formation, and glucose autoxidation, may be the cause of the elevated levels observed in GDM patients and diabetes patients. Elevated levels of lipids in GDM and diabetes patients can also result in oxidative stress because of increased mitochondrial uncoupling and β-oxidation, which increases ROS production and lipid peroxidation. An increase in ROS production and MDA levels has been reported in diabetic cases [6]. The production of free radicals and ROS by the placenta’s abundance of mitochondria may be the cause of the elevated MDA rate observed in both GDM participants and healthy pregnant women. The elevated serum MDA observed in this work is similar to reports that antioxidant levels are lower in pregnant women than in nonpregnant women because of the stress caused by gestation and increased placenta activity [28]. Antioxidants such as catalase (CAT), superoxide dismutase (SOD) and reduced glutathione (GSH) in the serum help to scavenge free radicals whose accumulation results in oxidative stress. Oxidative stress could arise as a result of increased placenta activity in GDM cases or auto-oxidation of glucose in DM situations. SOD activity, GSH and vitamin E levels were lower (*p* < in normal pregnant and diabetes patients but were high in nondiabetes nonpregnant women (Table 3). This could be a result of lipid peroxidation overwhelming the antioxidant system. This is further supported by the negative significant correlation at *p* < 0.01 between MDA and GSH observed in this study (Table S2). SOD activity was significantly lower (*p* < 0.05) in diabetes women than in gestational diabetes women, possibly because hyperglycemic conditions persist for a long time in diabetes women compared to GDM women who are pregnant during pregnancy. Decreased SOD, GSH and CAT activity in diabetes individuals compared to nondiabetes individuals has been reported [29, 30]. Expectant mothers could consume foods rich in antioxidants such as nuts and fruits to compensate for the oxidative stress caused by pregnancy.

In addition, Table 2 revealed that GDM patients had significantly greater serum TG and HDL-C levels (*p* < 0.05) than both normal pregnant individuals and nondiabetes nonpregnant individuals (Table S1). Moreover, the serum LDL-C levels of diabetes subjects were significantly greater (*p* < 0.05) than those of normal pregnant and nondiabetes subjects. This increase could be attributed to the fact that insulin resistance promotes the synthesis of lipids and fatty acids in GDM and diabetes women while suppressing lipolysis, which aligns with the findings of Ottaviani et al. [31]. The significant increase (*p* < 0.05) in lipoproteins cholesterol in GDM and diabetes women could be a result of increased cholesterol, it also suggests that gestation modifies lipoprotein levels. High cholesterol has been found to increase the need for LDL-C and HDL-C since both transport cholesterol throughout the body. Modulation of lipoproteins levels in GDM and DM states has been reported [32, 33]. Pregnant and nonpregnant women may also perform moderate physical exercise to burn excess fats (lipids), which may accumulate to cause insulin resistance, leading to or complicating diabetes mellitus conditions. These exercises will help in a long way and could also serve as palliative measures in the management of GDM and DM.

The Na^+^/K^+^ ATPase that pumps potassium ion (K^+^) and sodium ion (Na+) helps in the movement of glucose into the cells for metabolism. Bicarbonate ion (HCO_3_^−^) helps in regulating blood pH and diabetic acidosis has been associated with DM. The serum concentrations of K^+^ and HCO_3_^−^, as shown in Table 1, were significantly lower (*p* < 0.05) in GDM and diabetes women than in nondiabetes nonpregnant women. This decrease may be the consequence of these women’s diuresis and increased urine loss due to diabetes ketoacidosis. This finding is in line with that of Su et al., which also revealed that diabetes conditions are related to ketoacidosis [34]. These ions are reabsorbed in the kidney’s proximal tubule under normal physiological state [35]. The low levels of these ions also observed in normal pregnant women may be because, during pregnancy, the activity of the placenta generates reactive oxygen species, leading to lipid peroxidation, which compromises membrane integrity. This is supported by the work of Grossini et al. and Grzeszczak et al. who reported similar findings [36, 37]. This might result in Na+/K + ATPase malfunction in the cell membrane, which would change the electrolyte concentration outside of the cell. Osmotic diuresis caused by hyperglycemia in both GDM and diabetes women causes the loss of extracellular ions through the urine, which eventually lowers the concentration of ions in the circulatory system. Table S3 further confirms this finding by showing a significant negative correlation (-0.505) at *p* < 0.01 between FPG and Na^+^ and − 0.333^*^ at *p* < 0.05 between FBS and HCO_3_^−^ in this study, which implies that an increase in blood glucose will cause a decrease in Na^+^ and HCO_3_^−^ concentrations. Bicarbonate (HCO_3_^−^) was significantly (*p* < 0.05) lower in diabetes women than in nondiabetes nonpregnant women, possibly because of frequent urination by diabetes patients. However, glucose oxidation produces carbon IV oxide, which readily combines with water with the aid of carbonic anhydrase to form HCO3^−^ and is excreted in the urine.

Progesterone, prolactin and follicle-stimulating hormones (FSH) are female hormones associated with gestation. Additional progesterone and prolactin from the placenta in a pregnancy state could lead to glucose intolerance by inhibiting insulin action resulting in GDM [39]. There was an increase in the serum level of progesterone and prolactin in pregnant diabetes women compared to nondiabetes nonpregnant women (Table 5). This may be caused by the placenta, which is a further source of these hormones, as well as the high cholesterol and triglyceride (lipid) levels in the GDM group [32]. Progesterone and prolactin levels rise during pregnancy; these may act as agonists for insulin receptors, leading to insensitivity to glucose, as observed in GDM. It is also possible to biosynthesize progesterone via cholesterol, which is abundant in GDM patients. A class of enzymes known as cytochrome P450scc first obtains pregnenolone via cholesterol; pregnenolone is then subjected to a two-step reaction involving 3β-hydroxysteroid dehydrogenase and isomerase to generate progesterone [38]. Although progesterone is crucial for triggering the secretion of insulin in the early stages of pregnancy, as pregnancy progresses, elevated progesterone levels cause insulin resistance. Liu et al. likewise reported that oestrogen-related hormones lead to insulin dysfunction [39]. Follicle-stimulating hormone (FSH) levels were lower in both GDM and pregnant women (Table 5) than in diabetes and nondiabetes nonpregnant subjects because pregnancy stops ovulation and menstruation, for which FSH is needed [40]. There was no significant difference in the level of FSH between GDM patients and pregnant women or between diabetes and nondiabetes nonpregnant subjects; this suggests that diabetes condition does not affect FSH levels.

## Conclusion

This study suggested that pregnancy, diabetes and gestational diabetes conditions could cause a depletion of antioxidants and electrolytes, and modulate blood pressure, hormones and lipid profile levels in the women’s system. Though this could vary from one population or the other; this knowledge of these biochemical parameters will help suggest ways to address and manage GDM and DM thereby reducing complications associated with both disorders in pregnant and nonpregnant women respectively. Further investigation could be done on other biochemical parameters associated with glucose metabolism in both cases.

## Supplementary Information

Supplementary Material 1

Supplementary Material 2

Supplementary Material 3

## Data Availability

It will be made available on reasonable request.

## Abbreviations

ANOVA: analysis of variance
BMI: body mass index
FPG: fasting plasma glucose
1 HPP and 2 HPP: 1 h and 2 h postprandial
SPSS: Statistical Package for Social Sciences
TG: triglycerides
TC: total cholesterol
HDL-C and LDLC: high-density and low-density lipoprotein cholesterol
GSH: reduced glutathione
CAT: catalase
MDA: malonyl dialdehyde
SOD: superoxide dismutase
Vit: vitamin
Na^+^: sodium ion
K^+^: potassium ion
HCO_3_^−^: bicarbonate ion
