# Supplementary figures and images for: Effects of gestational diabetes mellitus and diabetes mellitus on lipid profile, antioxidants, hormones and electrolytes status in a population of Nigerian women
Source: Cardiovasc Diabetol Endocrinol Rep. 2025 Jan 21;11:1. doi: 10.1186/s40842-024-00206-4 (PMC11964098; doi:10.1186/s40842-024-00206-4)

**ETHICAL APPROVALS**


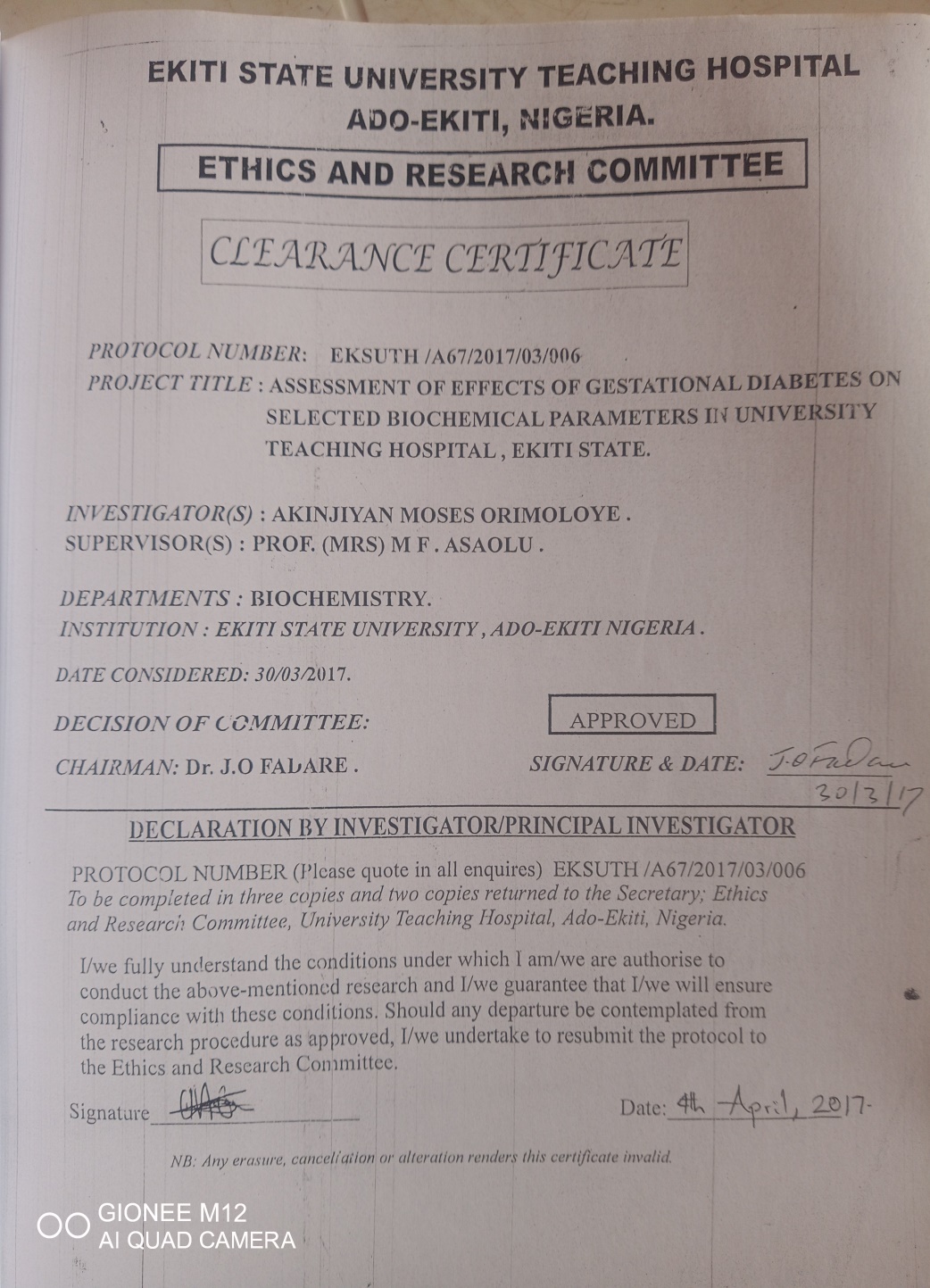


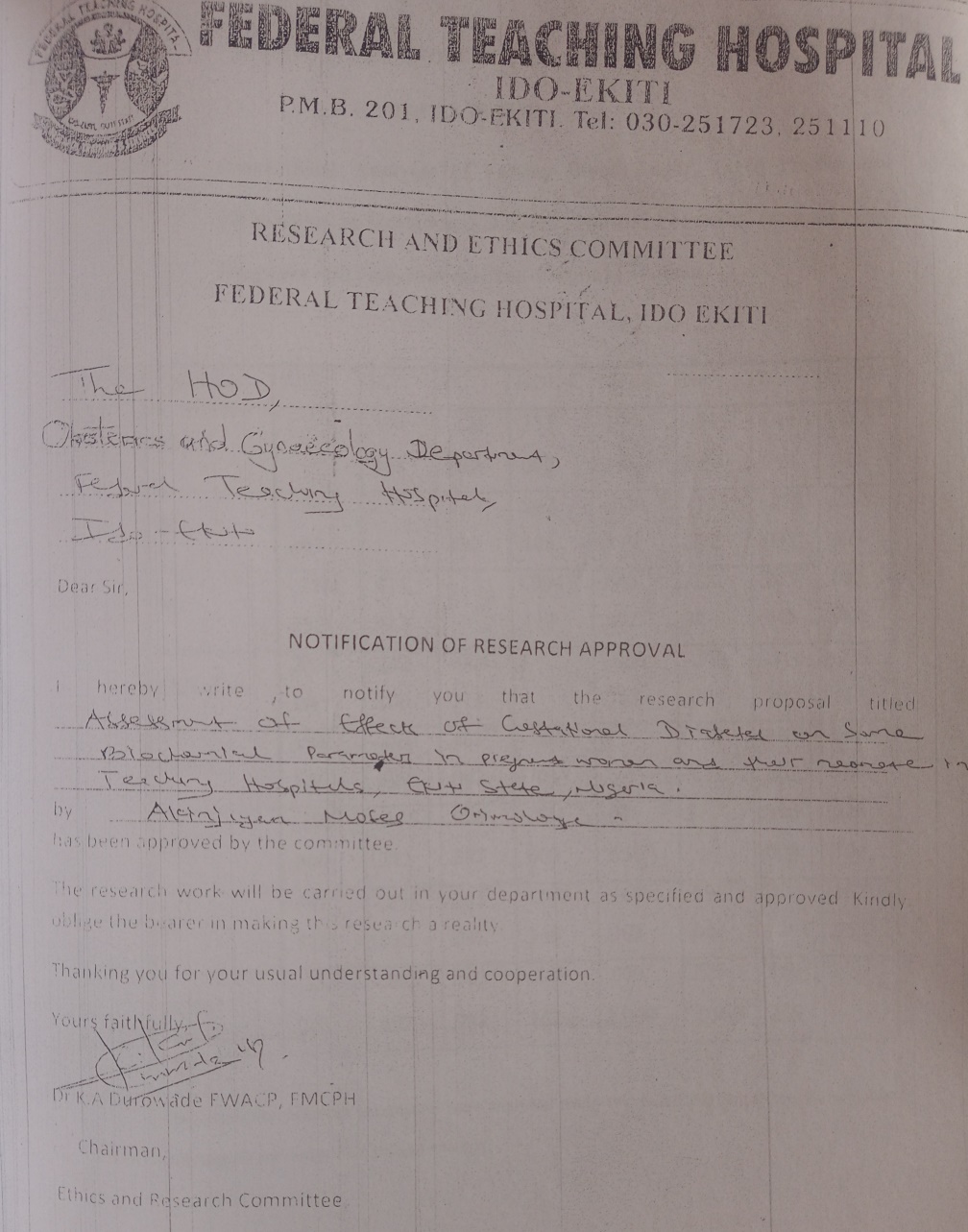

Supplement: Supplementary file 1 — Supplementary Material 1 [file 40842_2024_206_MOESM1_ESM.docx]
