# Supplementary material for: Effects of gestational diabetes mellitus and diabetes mellitus on lipid profile, antioxidants, hormones and electrolytes status in a population of Nigerian women
Source: Cardiovasc Diabetol Endocrinol Rep. 2025 Jan 21;11:1. doi: 10.1186/s40842-024-00206-4 (PMC11964098; doi:10.1186/s40842-024-00206-4)
Supplement: Supplementary file 2 — Supplementary Material 2 [file 40842_2024_206_MOESM2_ESM.docx]

**Supplementary Tables of Correlation analysis**

**Table S1: Correlation Analyses of Fasting Blood Sugar and Lipid Profile of gestational diabetic, normal pregnant, diabetic nonpregnant and nondiabetic nonpregnant women**

| Variables | | FPG | | TG | TC | HDL-C | LDL-C |
| --- | --- | --- | --- | --- | --- | --- | --- |
| FPG |  | | 1 | .194 | -.121 | .353^**^ | .194 |
|  |  | |  | .163 | .392 | .000 | .186 |
| TG |  | | .194 | 1 | .114 | .242 | -.020 |
|  |  | | .163 |  | .422 | .106 | .899 |
| TC |  | | -.121  .392 | .114  .422 | 1 | -.004  .113 | .463^**^  .002 |
| HDL-C |  | | .353^**^ | .242 | -.004 | 1 | -.066 |
|  |  | | .000 | .106 | .113 |  | .703 |
| LDL-C |  | | .194 | -.020 | .463^**^ | -.066 | 1 |
|  |  | | .186 | .899 | .002 | .703 |  |

The correlation between parameters was analyzed using a two-tailed Pearson R test for variables with significant levels *= 0.05 and **= 0.01. Key: FPG = Fasting plasma glucose, TG = Triglycerides, TC = Total cholesterol, HDL-C = High-density lipoprotein cholesterol and LDL-C = low-density lipoprotein-cholesterol.

**Table S2: Correlations analyses between Malonyldialdehyde and Antioxidants in gestational diabetic, normal pregnant, diabetic nonpregnant and nondiabetic nonpregnant women**

| Biochemical  variables | | MDA | GSH | CAT | SOD |
| --- | --- | --- | --- | --- | --- |
|  | |  |  |  |  |
| MDA |  | 1 | -.410^**^ | .116 | -.186 |
|  |  |  | .006 | .472 | .232 |
| GSH |  | -.410^**^ | 1 | .030 | .168 |
|  |  | .006 |  | .836 | .229 |
| CAT |  | .116 | .030 | 1 | -.160 |
|  |  | .472 | .836 |  | .263 |
| SOD |  | -.186 | .168 | -.160 | 1 |
|  |  | .232 | .229 | .263 |  |

The correlation between parameters was analyzed using a two-tailed Pearson R test for variables with significant levels *= 0.05 and **= 0.01. Key: GSH = Reduced glutathione, CAT = Catalase, SOD = Superoxide dismutase, MDA = Malonyldialdehyde.

**Table S3: Correlations Analyses of Fasting Blood Sugar and Electrolytes in gestational diabetic, normal pregnant, diabetic nonpregnant and nondiabetic nonpregnant women**

| Variables | | FPG | Na^+^ | K^+^ | HCO_3_^-^ |
| --- | --- | --- | --- | --- | --- |
| FPG |  | 1 | -.505^**^ | .041 | -.333^*^ |
|  |  |  | .000 | .773 | .015 |
| Na^+^ |  | -.505^**^ | 1 | .396^**^ | .228 |
|  |  | .000 |  | .004 | .104 |
| K^+^ |  | .041 | .396^**^ | 1 | .162 |
|  |  | .773 | .004 |  | .255 |
| HCO_3_^-^ |  | -.333^*^ | .228 | .162 | 1 |
|  |  | .015 | .104 | .255 |  |

.

The correlation between parameters was analyzed using a two-tailed Pearson R test for variables with significant levels *= 0.05 and **= 0.01. Key: FPG= Fasting plasma glucose, Na^+^ = Sodium ion K^+^ = Potassium ion and HCO_3_^-^ = bicarbonate ion.
