# Supplementary material for: Effects of gestational diabetes mellitus and diabetes mellitus on lipid profile, antioxidants, hormones and electrolytes status in a population of Nigerian women
Source: Cardiovasc Diabetol Endocrinol Rep. 2025 Jan 21;11:1. doi: 10.1186/s40842-024-00206-4 (PMC11964098; doi:10.1186/s40842-024-00206-4)
Supplement: Supplementary file 3 — Supplementary Material 3 [file 40842_2024_206_MOESM3_ESM.docx]

**EFFECTS OF GESTATIONAL DIABETES MELLITUS AND DIABETES MELLITUS ON LIPID PROFILE, ANTIOXIDANTS, HORMONES AND ELECTROLYTES STATUS IN A POPULATION OF NIGERIAN WOMEN**

Dear Reviewer,

The authors appreciate the efforts of reviewer 3 and the editor for his/her observations and efforts to improve the quality of this work. Though we effected some changes as in the earlier, we are re-examining our responses. These changes are highlighted in red in the manuscript. Thanks

Submission ID:  **CDEN-D-24-00046R2**

Thank you.

On behalf of the Authors,

Akinjiyan, Moses Orimoloye.

REVIEWER REPORTS

Reviewer #3: Authors are yet to effect the content of the initial review in the revised manuscript. The manuscript submitted and the point-by-point response seems to be at variance with each other. This should be quickly addressed. Also, the responses from the point-by-point responses still appear insufficient in some areas and need to be reconsidered.

Below are the issues raised

Title:
Effects of Gestational Diabetes Mellitus and Diabetes Mellitus on Lipid Profile, Antioxidants, Hormones and Electrolytes status among a Nigerian population
-       The above title should more appropriately read, "Effects of Gestational Diabetes Mellitus and Diabetes Mellitus on Lipid Profile, Antioxidants, Hormones and Electrolytes status in a population of Nigerian women."

**Response:** Thanks esteemed and distinguished reviewer, the title has been modified accordingly in the manuscript

Abstract:
-       At first glance, the abstract lacks a clear rationale, leaving the reader without an understanding of why this study is necessary. This is a significant issue. Without a clear reason, the study's relevance and contribution to the field are obscured, weakening the overall impact of the research.

**Response:** Thanks highly esteemed reviewer; the study aims to evaluate the effect of GDM and DM on biochemical parameters such as lipids, antioxidants, electrolytes and hormones. We wish to drive home that apart from blood sugar, fluctuations in the level of these parameters could help detect and manage diabetic conditions in pregnancy while could help prevent further complications associated with both disease statuses. This we have vividly stated in the manuscript also.

-       The methods section in an abstract should provide basic information on how the study was conducted. Missing details like study design, statistical methods etc., make it difficult to assess the quality of the findings.

**Response:** Thanks esteemed reviewer, the abstract has been rewritten: The study is a cross-sectional design and followed convenience sampling in four groups (n=50). Results were analysed using ANOVA and p<0.05 was used as the level of significance difference.

-       The first use of abbreviations should be accompanied by the full term (e.g., Superoxide dismutase (SOD)), and this was not consistently done. This is a basic but crucial element of scientific writing.

**Response:** Thanks esteemed reviewer, the list of abbreviations was given at the end of the abstract because of the limited number of words permitted for the abstract

-       The conclusion does not effectively make sense of the study's findings. A strong conclusion should tie the results back to the study's aims, offering insights or implications of the findings.

**Response:** Thanks esteemed reviewer, the conclusion has been rewritten

Introduction:
-       Use of the term "Introduction" is more appropriate as presenting the background of the study, highlighting the problem to be addressed, proffering a hypothesis, and stating the aim can be effectively captured here.

**Response:** Thanks esteemed reviewer, “background” has been replaced with “introduction”

-       This section is thorough and attempted to cover all the biological aspects that might be investigated in the study. However, it could be improved by organizing the content in a more logical flow, starting with the broader context of GDM and DM, and then narrowing down to specific biological factors like oxidative stress, lipids, electrolytes, and hormones that are directly related to the research question. Each paragraph should build upon the previous one, leading to a clear rationale for the study.

**Response:** Thanks esteemed reviewer, this has been worked on. The introduction is now more elaborate.

-       The presentation of the prevalence data is somewhat scattered, and there's a lack of consistency in the reporting of statistics. For instance, mentioning that "the reported prevalence is 2.98 per 1000 pregnancies, and 1.7% of expectant mothers". What does this mean? Are these figures referring to the same population or different studies? Also, this statement does not reflect the current realities of increasing global and local (Nigeria) prevalence of GDM. Authors may access recent publications like doi: 10.4314/ahs.v23i4.37 and doi: 10.4103/ijem.ijem_301_21 for the current prevalence of the disease.

**Response:** Thanks esteemed reviewer, we have checked these two publications and improved on these statistics. It has been rewritten thus and appropriately referenced.

“In Africa, the prevalence of GDM in the antenatal population is low and varies; the reported prevalence is 3.7% of expectant mothers while 13.9% prevalence was found in Nigeria”

-       The sentence "Women who develop type 1 DM during pregnancy or who have undetected type 2 DM that is known during pregnancy are referred to as having GDM" is misleading. GDM specifically refers to glucose intolerance first recognized during pregnancy, not pre-existing type 1 or type 2 diabetes. Presently, a newer definition can be accessed here; doi: 10.2337/dc24-S002.

**Response:** Thanks esteemed reviewer, we have rephrased the statement, “Women who develop type 1 or type 2 DM during pregnancy are referred to have gestational diabetes mellitus”

-       This introduction section failed to clearly highlight the problem which the study aims to solve, make any hypotheses, justify the study or state the objectives of the study.

**Response:** Thanks, the objectives have been stated at the tail end of the introduction. The problem statement is complications associated with diabetes mellitus in pregnancy.

Materials and Methods:

The "Materials and Methods" section covers many of the necessary elements but could benefit from additional detail and clarification to enhance transparency and reproducibility.

**Response:** We are grateful for your comments sir/ma. Additional methods for the procedures have been added.

-       The absence of a clear description of the study design makes it difficult to assess the validity of the results or compare the findings with those from other studies. The authors should explicitly state the study design (e.g., cross-sectional, case-control, or cohort) and provide a rationale for why this design was chosen for the study's objectives.

**Response**: Thanks esteemed reviewer, the study employed is a cross-sectional study and this has been included. This method could be employed for public health investigations.

-       The authors should describe how participants were selected including the sampling method (e.g., convenience sampling, stratified sampling) and any random assignment to groups.
-       If the study involved data collection over a specific period, this should be mentioned. Specify the duration of the study.

**Response:** Thanks, the sampling method is convenience sampling and the duration of the study is from 2016 to 2017

-       What was the basis for the chosen sample size and how was this determined?

**Response:** Dear reviewer, the following formula was used: 𝑁=	𝑡^2^	𝑃 (1−𝑃)

𝑚^2^

Where;

𝑁: sample size,

𝑡: confidence interval of 95% (standard value of 1.96),

𝑃: prevalence rate (3.5%),

𝑚: margin of error (standard value of 0.05).

Hence 𝑁 **=** 54.75. An approximate sample size of 50 per group and 200 (4 groups) was chosen for this study.

-       In the exclusion criteria section, the criteria for excluding participants based on other medical conditions should include the rationale for using these conditions.

**Response:** Thanks, the rationale is that these medical conditions could influence the biochemical parameters being examined. It has been included.

-       Concerning the reporting on the analysis section, a more detailed account of the analytical procedures would strengthen the section significantly. For instance, in 2.3.1, the authors wrote, "Lipid peroxidation (malonyldialdehyde) and antioxidant (SOD, GSH and CAT) levels were determined using standard procedures as described previously [17]. Estimates of the concentrations of triglycerides, high- and low-density lipoprotein cholesterol, and serum total cholesterol were made following the methods of [18]". This is an incomplete and unacceptable reporting format. Authors should clearly and concisely describe the principle of the test method utilized. The same should be applied to other aspects as in 2.3.2 and 2.3.3

**Response:** Thanks respected reviewer, the procedures have all been well-described for all the parameters investigated.

-       The absence of a clear description of the basis for categorising participants into the various groups present a major methodological flaw. What criteria was used to diagnose GDM, DM etc.

**Response:** Thanks reviewer; Pregnant and non-pregnant women with fasting plasma glucose greater than 5.56 mmol/l were diagnosed with GDM (group 1) and DM (group 3) respectively after 1 hr and 2 hrs, 75 g oral glucose tolerance test was done. It has been included in the manuscript.

-       The authors should provide a clearer rationale for the choice of statistical methods.

**Response:** Thanks distinguished reviewer, one-way analysis of variance and P<0.05 as the level of significance has been widely used and reported for this kind of study, hence our choice which is based on this literature using SPSS version 20.

Results:
This section provides detailed findings from the study. It is well-organized with clear subheadings that correspond to different aspects of the study's results (e.g., anthropometric parameters, glucose and lipid profiles, antioxidant activity, etc.). This helps the reader easily navigate through the results, but some areas could be improved for clarity, organization, and overall scientific rigor.

-       The results are presented as mean ± SD, which is appropriate for continuous variables. However, the text occasionally mixes up how it presents these values, sometimes stating the mean first and other times the standard deviation. Consistency in this format would improve readability.

**Response:** Thanks esteemed reviewer for pointing this out; we have rectified this. The standard we conformed with is the mean first, then followed by the standard deviation.

-       The section frequently mentions when differences are statistically significant (p<0.05), which is crucial. However, the use of "p>0.05" when no significance is found (as in the hormone levels) is not always necessary.

**Response:** Thanks for pointing this out dear reviewer; we are sorry for this and corrected it.

Discussion:
The discussion effectively interprets the study's findings and links them to existing literature, but it would benefit from a more critical analysis of potential confounding factors, clearer explanations, consideration of clinical implications, and a discussion of study limitations.

**Response:** Thanks respected reviewer, we have added flesh to the discussion.

-       A large portion of the discussion were merely a repetition of the result findings instead of a discussion of its implications.

**Response:** Thanks esteemed reviewer, the discussion has tried to explain possible reasons and implications for the results observed in the study

-       The authors mentioned genetic and environmental factors as potential contributors to GDM but did not delve into the possibility of confounding factors influencing the results. For example, other variables like diet, physical activity, or socioeconomic status might have impacted the outcomes and should be considered in the analysis.

**Response:** Thanks esteemed reviewer, we have included this sir/ma. Grateful

-       Insertion of references do not make or incomplete expression of ideas. The authors expect readers to access each referenced article to make sense of each sentence while reading, and that is unacceptable!

**Response:** Thanks respected reviewer, we have adjusted this sir.

-       In the first paragraph, the authors wrote, "The buildup of adipose tissues or fats, such as visceral fats, is typically what leads to obesity (liver, muscle and other organs). Environmental factors such as overfeeding or genetics may be responsible for this fat buildup [24] (Qualls-Creekmore et al., 2020)". It is unusual to use both the Vancouver and another referencing method in one article.

**Response:** Thanks esteemed reviewer for pointing this out; we have corrected this

    Paragraph 3 reads, "Table 2 shows that the mean fasting plasma glucose levels of gestational diabetes and diabetes women were significantly greater (p < 0.05) than those of normal pregnant and nondiabetes nonpregnant women, respectively". The authors could consider omitting comparing the FBG across these groups since these groups (at least the DM groups and to an extent the GDM group) were most likely categorized based on same FBG. It is as counter-intuitive as comparing BMI categories (underweight, normal, overweight, obese) by their BMI scores.

**Response:** Thanks reviewer, the statement has been removed.

-       In some instances, the discussion could be clearer. For example, when discussing the correlation between malonyldialdehyde (MDA) levels and oxidative stress, it would be helpful to explain more explicitly how the study's results align or differ from previous research. Additionally, some sentences are overly complex and could be simplified for better readability.

**Response:** Thanks esteemed reviewer, we have made it simpler and compared it to previous research. This is highlighted in red and appropriately referenced.

“Elevated levels of lipids in GDM and diabetes patients can also result in oxidative stress because of increased mitochondrial uncoupling and β-oxidation, which increases ROS production and lipid peroxidation. An increase in ROS production and MDA level has been reported in diabetic cases [6].”

-       Crucially, the discussion does not fully explore the clinical implications of the findings. For instance, how might the observed increases in BP, fasting plasma glucose, and lipid profiles inform treatment strategies for GDM or diabetes? Discussing the potential impact on patient management could add depth to the discussion.

**Response:** We are grateful for this, this has been included in the discussion and conclusion e.g. “Increased physical activity and limiting fatty food intake could help reduce lipid buildup, and thus manage DM both with and without pregnancy”.

-       While the discussion cites relevant literature, it could benefit from a more thorough comparison with previous studies. For example, how do the findings regarding lipid profiles in GDM and diabetes women compare with those in other populations? Addressing this could provide a more comprehensive understanding of the results.

**Response:** Thanks reviewer, we do compare the results with other literatures. We have done that of the lipid too that was pointed out. E.g. Modulation of lipoproteins level in GDM and DM states have been reported.

Dalfrà, M. G., Burlina, S., Ragazzi, E., Pastrolin, S., Sartore, G., & Lapolla, A. (2024). Lipid profile in women of different ethnicity with gestational diabetes: Relationship with fetal growth. Journal of diabetes investigation, 15(3), 355–363. https://doi.org/10.1111/jdi.14124

-       The discussion does not address potential limitations of the study, such as sample size, potential biases, or the generalizability of the findings. Acknowledging these limitations would provide a more balanced perspective and improve the credibility of the conclusions.
-       The recommendations should be incorporated into the discussion section without making it a separate heading.

**Response:** Thanks reviewer, we have done as suggested, recommendations have been input into the discussion and the conclusion written with some limitations embedded. Gracias

Conclusion:
-       The conclusion section provides a basic summary of the study's findings but lacks depth in discussing the implications, addressing study limitations, and situating the results within a broader context. Improving clarity, precision, and providing a more detailed interpretation of the findings would enhance the overall effectiveness of the conclusion.

-       The conclusion failed to wrap up the study as it should for obvious reasons. A better approach would have been to:

o       Firstly, highlight the main problem of the study and identify the research gap. Then link these with the aim of the study.

o       Secondly, outline your key findings and state its implications for clinical practice.
o       Finally, highlight the major limitations and proffer recommendations for future research.

**Response:** Thanks reviewer, the conclusion has been rewritten to address the issues raised. Our conclusion is below.

**Conclusion**

This study suggested that pregnancy, diabetes and gestational diabetes conditions could cause a depletion of antioxidants and electrolytes, and modulate blood pressure, hormones and lipid profile levels in the women’s system. Though this could vary from one population or the other; this knowledge of these biochemical parameters will help suggest ways to address and manage GDM and DM thereby reducing complications associated with both disorders in pregnant and nonpregnant women respectively. Further investigation could be done on other biochemical parameters associated with glucose metabolism in both cases.

Summary:
Although there were no obvious methodological flaws, possibly due to poor reporting, this study has failed to meet the basic requirements of academic writing and will require a complete overhaul to enable its consideration for publication in a reputable journal like Clinical Diabetes and Endocrinology.

**Response:** Thanks esteemed reviewer, we have re-written and thoroughly edited the manuscript to address this concern.
